# TRIM16 Acts as an E3 Ubiquitin Ligase and Can Heterodimerize with Other TRIM Family Members

**DOI:** 10.1371/journal.pone.0037470

**Published:** 2012-05-21

**Authors:** Jessica L. Bell, Alena Malyukova, Jessica K. Holien, Jessica Koach, Michael W. Parker, Maria Kavallaris, Glenn M. Marshall, Belamy B. Cheung

**Affiliations:** 1 Children’s Cancer Institute Australia for Medical Research, Lowy Cancer Research Centre, University of New South Wales, Randwick, New South Wales, Australia; 2 St Vincent’s Institute of Medical Research, Fitzroy, Victoria, Australia; 3 Department of Biochemistry and Molecular Biology, Bio21 Molecular Science and Biotechnology Institute, The University of Melbourne, Parkville, Victoria, Australia; 4 Centre for Children’s Cancer and Blood Disorders, Sydney Children’s Hospital, Randwick, New South Wales, Australia; Institute of Enzymology of the Hungarian Academy of Science, Hungary

## Abstract

The TRIM family of proteins is distinguished by its tripartite motif (TRIM). Typically, TRIM proteins contain a RING finger domain, one or two B-box domains, a coiled-coil domain and the more variable C-terminal domains. TRIM16 does not have a RING domain but does harbour two B-box domains. Here we showed that TRIM16 homodimerized through its coiled-coil domain and heterodimerized with other TRIM family members; TRIM24, Promyelocytic leukaemia (PML) protein and Midline-1 (MID1). Although, TRIM16 has no classic RING domain, three-dimensional modelling of TRIM16 suggested that its B-box domains adopts RING-like folds leading to the hypothesis that TRIM16 acts as an ubiquitin ligase. Consistent with this hypothesis, we demonstrated that TRIM16, devoid of a classical RING domain had auto-polyubiquitination activity and acted as an E3 ubiquitin ligase *in vivo* and *in vitro assays*. Thus via its unique structure, TRIM16 possesses both heterodimerization function with other TRIM proteins and also has E3 ubiquitin ligase activity.

## Introduction

The tripartite motif (TRIM)/Ring finger, B box, coiled-coil (RBCC) family of proteins are defined by the presence of the tripartite motif. The TRIM protein family is composed of more than 70 highly conserved proteins which have been implicated in a diverse range of biological processes including development, cell growth, differentiation, innate immune functions and cancer [1], [2]. One poorly characterized member of this family is TRIM16. TRIM16 or estrogen-responsive B box protein (EBBP) is a positive transcriptional regulator of the retinoic acid receptor β_2_ (RARβ_2_) gene [3], [4]. TRIM16 overexpression also enhances retinoid-induced differentiation, reduces neuroblastoma cell growth, migration, and significantly reduces tumorigenicity *in vivo* [5].

Typically, TRIM proteins contain a Really Interesting New Gene (RING) finger domain, one or two B-box domains, a coiled-coil domain and the more variable C-terminal domains. The RING and B-box domains are both cysteine-rich and bind zinc atoms, indicating possible interactions with proteins, DNA and RNA. The RING domain has E3 ubiquitin-ligase potential and can transfer ubiquitin to RING proteins as well as heterologous substrates [1], [2]. However, little is known about the function of B-box domains, although they occur widely in nature. Recently, the first solved structure of a B-box domain displays a structure similar to a RING domain, suggesting these domains evolved from a common ancestor [6], [7]. TRIM16 does not have a RING domain but does harbour two B-box domains, leading to the hypotheses that TRIM16 has RING-like domains and functions as an ubiquitin ligase. TRIM16’s C-terminus contains an RFP-like or B30.2/SPRY (B30.2) domain.

Several members of the TRIM protein family are known to homodimerize and heterodomerize with other TRIMs, and the coiled-coil domain is generally required for these functional associations [8], [9]. In this study, we show that TRIM16 can homodimerize through its coiled-coil domain but also bind to other TRIM proteins; Midline-1 (MID-1/TRIM18), Promyelocytic leukemia protein (PML/TRIM19) and TRIM24 (TIF-1α). Additionally, our molecular modelling of TRIM16’s B-boxes suggests zinc binding capability and we present evidence that B-box domains have ubiquitination capability, leading to the suggestion that other B-box-containing proteins may perform a similar function.

**Figure 1:**
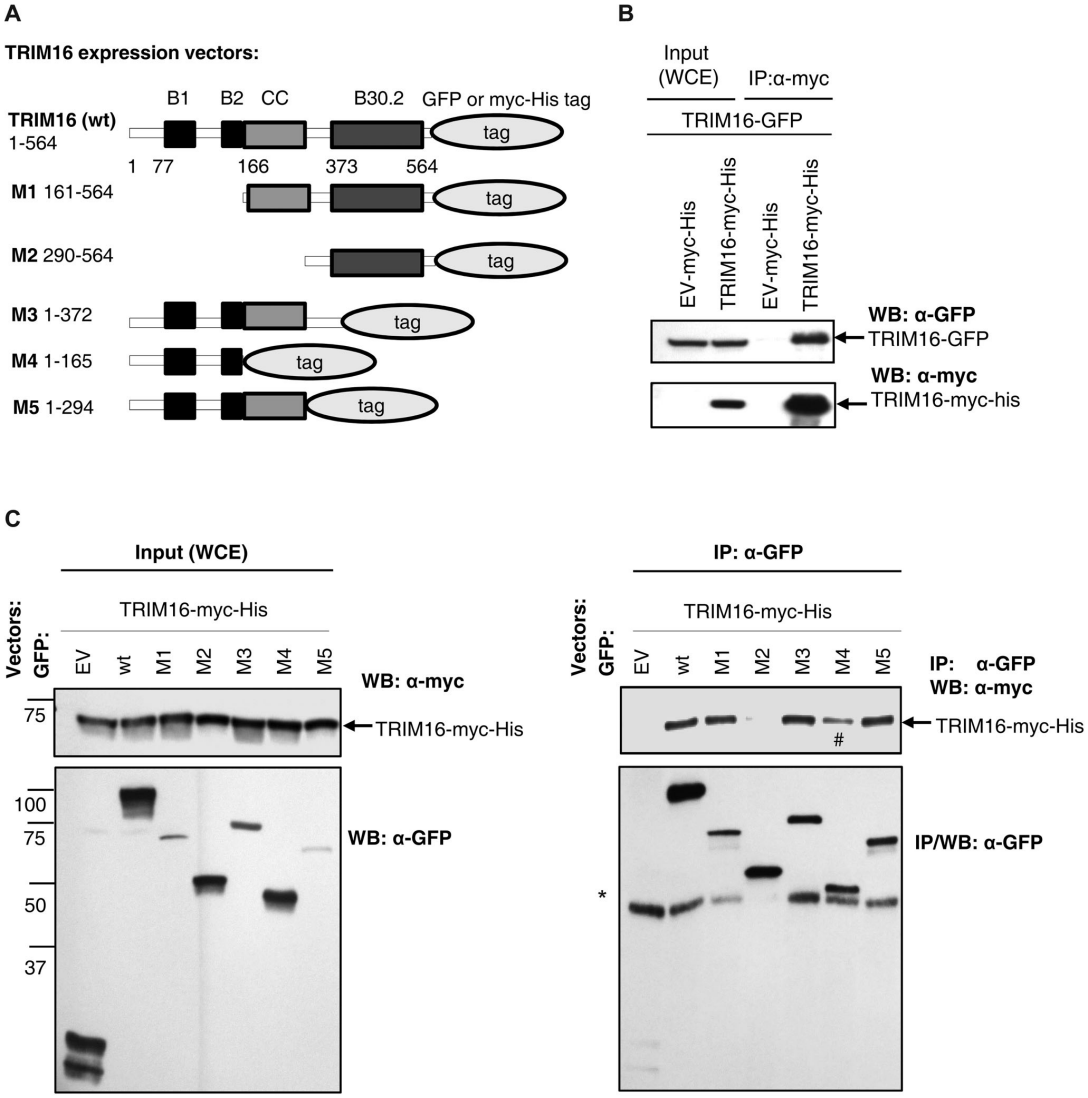
TRIM16 homodimerizes through its coiled-coil domain . (A) TRIM16-GFP domain deletion plasmids. (B) Co-transfection of TRIM16-GFP and TRIM16-myc-His in HEK293 cells and subsequent immunoprecipitation by anti-myc antibody (Ab) and Western blot with anti-GFP antibody. Whole cell extract (WCE) used as total input. (C) TRIM16 homodimerizes through its coiled-coil domain. GFP deletion mutants were co-transfected with the TRIM16-myc-His vector. Anti-GFP antibody was used to pull down proteins binding the GFP tagged proteins and the TRIM16-myc-His was used to detect self-association via its different tag (right panel). Transfection efficiency was confirmed (left panel). TRIM16-GFP mutants were efficiently pulled down (middle panel). * non-specific bands, # refer to text.

## Results and Discussion

### TRIM16 Homodimerizes via Its Coiled-Coil Domain

In the large and evolutionary conserved TRIM family, specific domains tend to operate as functional units. Many TRIM proteins homodimerize via their coiled-coil domains [2], [8], [9]. To further investigate the function of TRIM16, we tested whether TRIM16 is able to homodimerize. Two differently tagged TRIM16 expression vectors and 5 different deletion mutants of TRIM16 were used (Figure 1A); TRIM16-GFP and TRIM16-myc-his with both tags being fused separately to the C-terminus of the TRIM16 protein. The full-length TRIM16 and 5 deletion constructs were used to probe for the role of various domains in protein function (Figure 1A). HEK293 cells were co-transfected either empty vector pCMV6-AC-GFP and TRIM16-GFP or empty vector (EV) pCDNA-myc-His and TRIM16-myc-His. Immunoprecipitation of the myc-tag strongly indicated that TRIM16-GFP and TRIM16-myc-His were homodimerizing (Figure 1B). We confirmed these results by using the anti-GFP antibody (Ab) for immunoprecipitation, and investigating whether GFP-tagged deletion mutants of TRIM16 were able to retain homodimerization with the TRIM16-myc-His. GFP fusion mutants bearing coiled-coil domains (mutant 1, 3 and 5) were able to homodimerize with the TRIM16-myc-His protein, indicating this region is required for TRIM16 homodimerization (Figure 1C and 1A). However a weak band (Lane 6 in Figure 1C) indicates that B-boxes are able to form some homodimers to a lesser extent.

**Figure 2:**
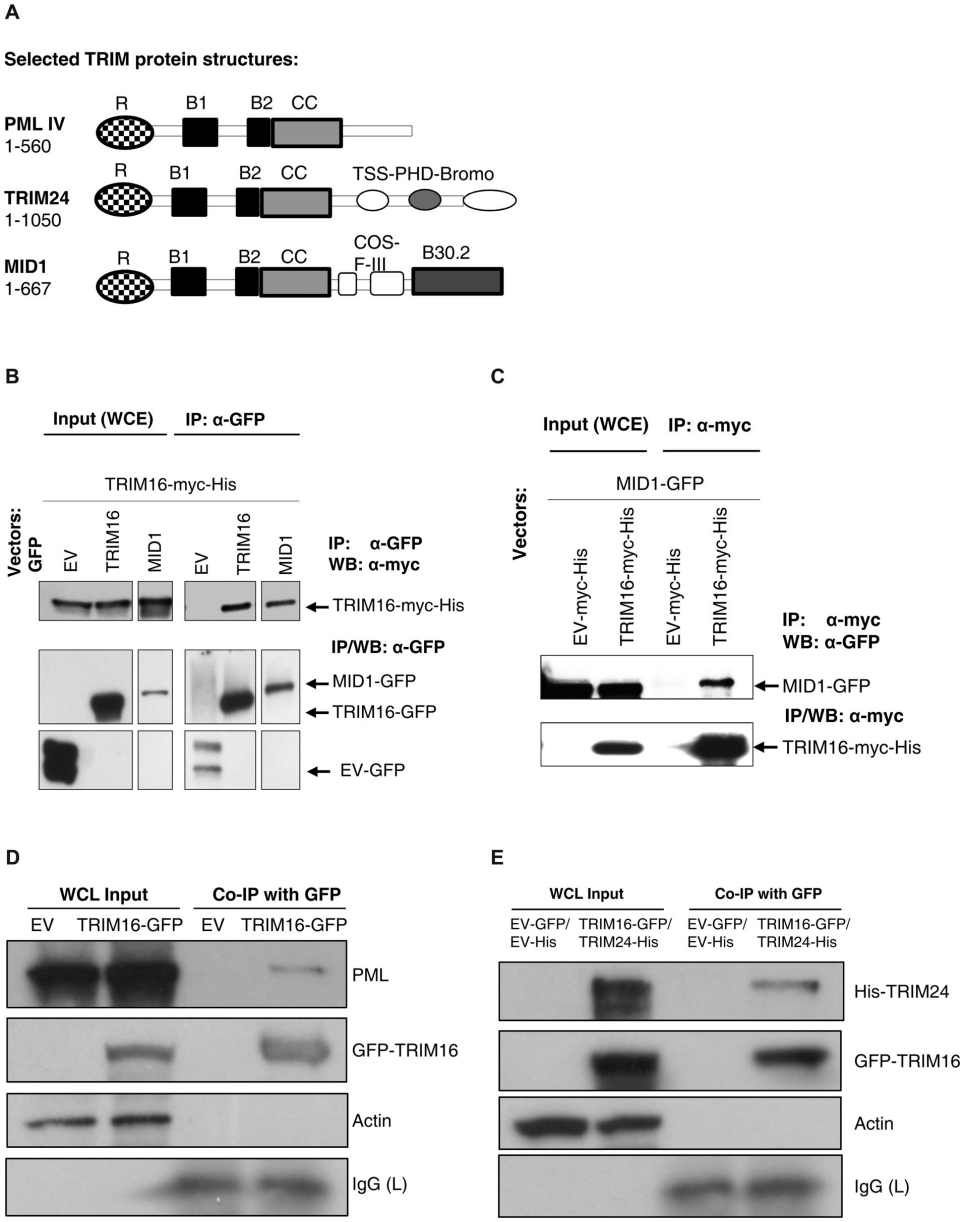
TRIM16 can heterodimerize with MID1, TRIM24 and PML. (A) Schematic structures of TRIM proteins used in heterodimerization studies. (B) TRIM16 binds MID1. Co-transfection of MID1-GFP and TRIM16-myc-His in HEK293 cells and subsequent immunoprecipitation by anti-myc antibody and Western blot with anti-GFP antibody. (C) MID1 was pulled down via its GFP antibody and a Western blot was performed to detect TRIM16-myc-His in the immunoprecipitated protein complex. (D) Whole cell lysates (WCL) of HEK293 cells transfected with empty vector (EV) or TRIM16-GFP were immunopreciptated with anti-GFP antibody. An anti-PML antibody was used to detect PML as a binding partner of TRIM16. (E) Lysates containing both TRIM16-GFP and TRIM24-His proteins were immunopreciptated with anti-GFP antibody. Anti-His antibody was used to detect the presence of TRIM24 in the TRIM16-associated complex.

### TRIM16 Heterodimerizes with MID1, TRIM24 and PML

TRIM proteins have been shown to interact with other TRIM family members. For example, TRIM24 is known to bind PML [10] and RET finger protein (RFP/TRIM27) through the B-box and coiled-coil domains [11]. Therefore it was important to establish whether TRIM16 may also form hetero-complexes. PML, TRIM24 and MID1 (Figure 2A) have E3-ligase ability through their RING domain and generally homo/heterodimerize through their coiled-coil domains [2], [8], [9]. To determine whether MID1 could interact with TRIM16, we performed co-transfection of MID1-GFP and TRIM16-myc-His in HEK293 cells and subsequent performed immunoprecipitation by anti-myc antibody and western blot with anti-GFP antibody (Figure 2B). Co-immunoprecipitation confirmed that TRIM16 and MID1 formed a complex in HEK293 cells (Figure 2B). The specificity of this interaction was validated by MID1 immunoprecipitation with an anti-GFP tag antibody and Western blot analyzed by myc-tag antibody, probing for TRIM16-myc-His (Figure 2C). The interaction with MID1 suggested that TRIM16 may have a function in the cytoskeleton, as MID1 associates with the microtubules and the cytoskeleton [12]. Interestingly, TRIM16 has been previously shown to bind the cytoskeletal protein; vimentin [5], further suggesting TRIM16 may has a role in the cytoskeleton function.

**Figure 3:**
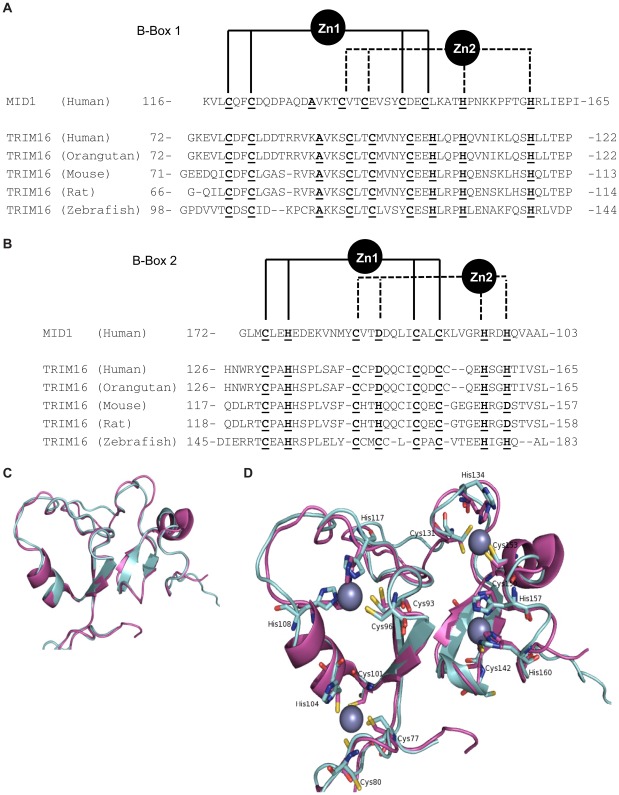
Amino acid sequence comparison of TRIM16 and MID1 and modeling of TRIM16 B-boxes. The conserved residues in the zinc binding regions are in bold underlined type and are; cysteine; C, histidine; H, alanine; A aspartic acid; D. (**A**) TRIM16 and MID1 share the zinc binding consensus sequence for B-box1. (B) TRIM16 and MID1 share the Zinc binding consensus sequence for B-box2. (C/D) Modeling of B-boxes reveals zinc binding capability. Superimposition of the alpha-carbon backbone of the B-boxes from the MDM1 NMR structure (purple) and the homology model of TRIM16 (blue-grey). These two structures overlay with an average root-mean-square deviation of 0.4 Å.

Other TRIM proteins, TRIM24 and PML also displayed binding with TRIM16 (Figure 2D and 2E). TRIM16-GFP was immunoprecipitated via its GFP tag, and the resulting associating proteins were subjected to western blot and probed for PML (anti-PML antibody) or TRIM24-His (anti-His antibody). In addition to the interaction observed with MID1, the interactions with TRIM24 and PML proteins demonstrate that TRIM16 is able to heterodimerise with at least three different classes of TRIM proteins.

TRIM16 is known to bind the promoter region of the RARβ gene [3]. Interestingly, TRIM24, also a transcription factor, has been shown to act in a complex with PML and the RARα and RXR receptors, which are known to bind the same promoter region sequence of RARβ as TRIM16 [10], [13]. PML is a tumour suppressor which has a significant role in the cell-cycle, differentiation and apoptosis. PML is required for the formation of large protein complexes called PML-bodies which are believed to have a role in transcription. PML and TRIM24 do not possess a B30.2 domain which suggests TRIM16’s heterodimerization is through the other domains on these proteins.

### TRIM16’s B-Boxes Possess RING-Like Folds

The solution structure of MID1’s B-boxes reveals they adopt RING-like folds with a modelled capacity for zinc-binding and possibly ubiquitination activity [7], [14]. We hypothesized that the B-boxes of TRIM16 also have RING-like folds. An amino acid sequence alignment of the human MID1 sequence with TRIM16 from various species was performed (Sim, ExPASy, CA). There was 46% sequence identity between the human B-box1 sequences of the two proteins (Figure 3A). TRIM16 shares the zinc binding consensus sequence to MID1 with the exception of the 6^th^ cysteine residue of the MID1 B-box1’s consensus sequence being substituted with a histidine residue in TRIM16. Other TRIMs (eg.TRIM29 (ataxia-telangiectasia group D complementing gene) and TRIM25 (The estrogen-responsive gene/EFP) also share this particular substitution [6]. An unbiased blast search of the NCBI database with TRIM16’s B-box1 sequence yields significant sequence identity with human TRIM16, TRIM29, TRIM25, MID1, RFP and MID2 (only TRIMs resulted, in order of significance all with E-values <0.009, and scores above 37.7). Analysis of TRIM16’s B-box2 sequences yielded similar results to B-Box1 (Figure 3B), although with a lower human sequence identity (28%) to MID1 (Sim, ExPASy, CA). When comparing the full-length of the sequence, TRIM16 shares low sequence identity with other TRIMs such as TRIM29, TRIM25, RFP, MID1 and MID2, except in the B-box1 region where the high homology amongst these TRIMs suggests a subgroup of TRIMs having a specific zinc-binding role in homeostasis.

**Figure 4:**
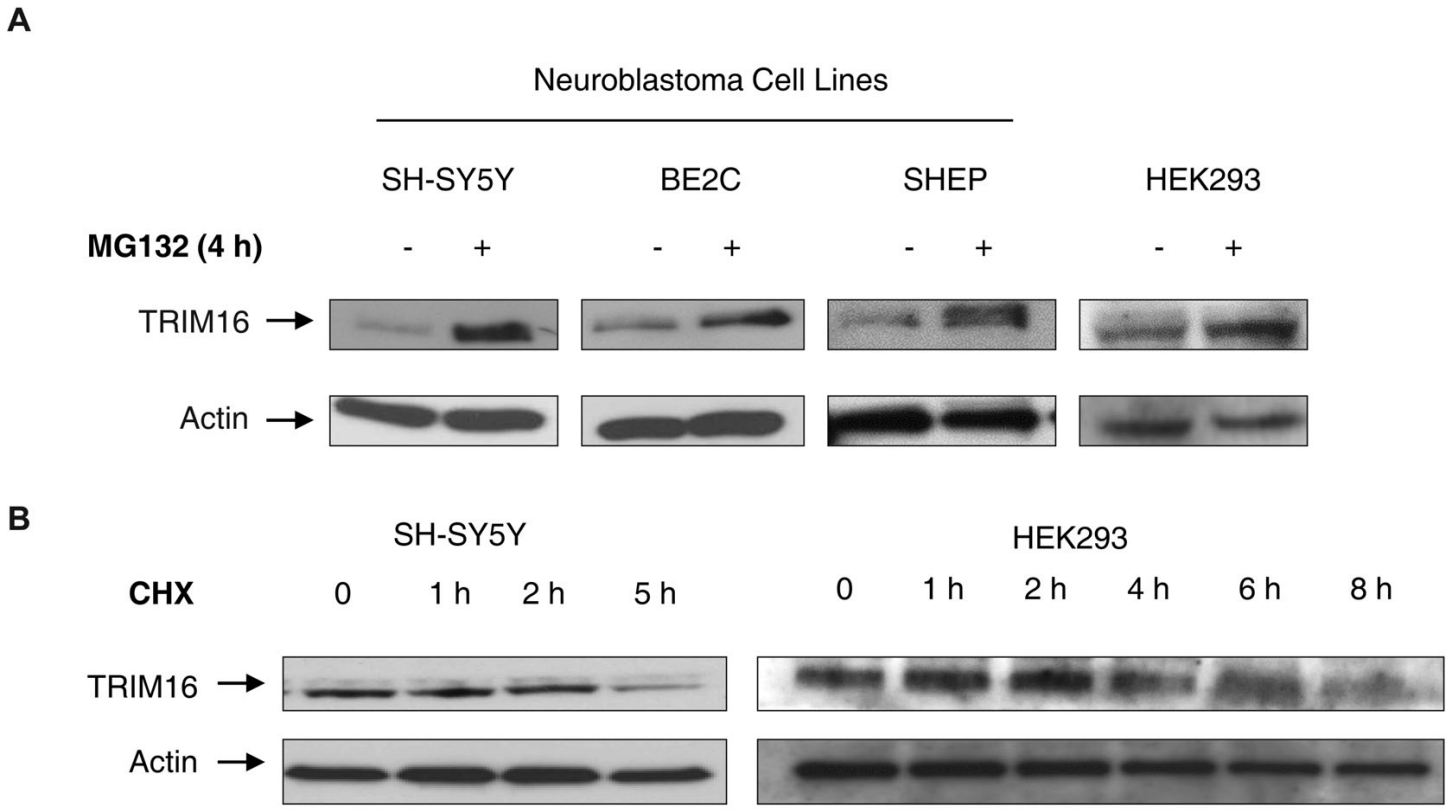
TRIM16 is an unstable protein regulated by the ubiquitin-proteasome system. (A) *In vivo* degradation assay was performed with cells being incubated with and without 30 µM MG132 for 4 hours. Lysates were subjected to Western blot analysis of endogenous TRIM16 antibody. (B) The liable nature of TRIM16 was further evaluated by inhibition of protein synthesis using 100 µg/ml cycloheximide. At the specified time points the cells were harvested and the protein were extracted for analysis by Western blots.

To gain greater insight into the structure of TRIM16, the amino acid sequence for human TRIM16 was threaded onto the top ten protein structure templates as predicted by the HHpred program (http://toolkit.tuebingen.mpg.de/). A homology model of the whole of TRIM16 was then constructed using MODELLER [15]. The homology model predicted a structured region for the B-Box domains; (Cys77 to His117 and Cys131 to His160) and the B30.2 domain (Pro379 to Leu536). The coiled-coil region could not be modelled, as no suitable templates existed. The B-box region quality of the model was confirmed by the PROCHECK program [16] which showed >97% of residues had backbone geometry falling in the favourable regions of the Ramachandran plot. Furthermore, superimposition of the B-box domains of TRIM16 onto the NMR solution structures of MID1 B1B2, not in the structure templates used for homology modeling, gave a very close overlay with an average α-carbon root-mean-square deviation of 0.4 Å (Figure 3C). TRIM16’s B-Boxes form an unusual “cross-brace” arrangement or “Intermolecular Ring Heterodimers” capable of zinc binding [14], [17], [18]. For Zn1, the coordinating residues are Cys77, Cys80 and His104; for Zn2, the residues are Cys86, Cys96, His 108 and His 117; for Zn3 they are Cys131, His134, Cys150 and Cys153; and for Zn4 the residues are Cys142, His157 and His160 (Figure 3D). The RING domain and B-box domains may share an ancestral motif [7], [14]. TRIM16 is one of the oldest TRIMs [19], yet is the only functionally studied TRIM with a B1-B2-CC-B30.2 motif, indicating the evolution of a RING-less TRIM is an ancient and biologically required phenomenon.

### TRIM16 Auto-Ubiquitinates via Its B-Boxes

Several TRIM proteins with RING domains have been implicated in the proteolysis, acting as E3 ubiquitin ligases. However, B-boxes are required for the E3 ligase ability of PML and its ability to transfer the small ubiqutin-like modifier (SUMO) from Ubc9 to p53 [20]. Similarly, MID1’s B-boxes enhance the E3 activity of its RING domain [21].

**Figure 5:**
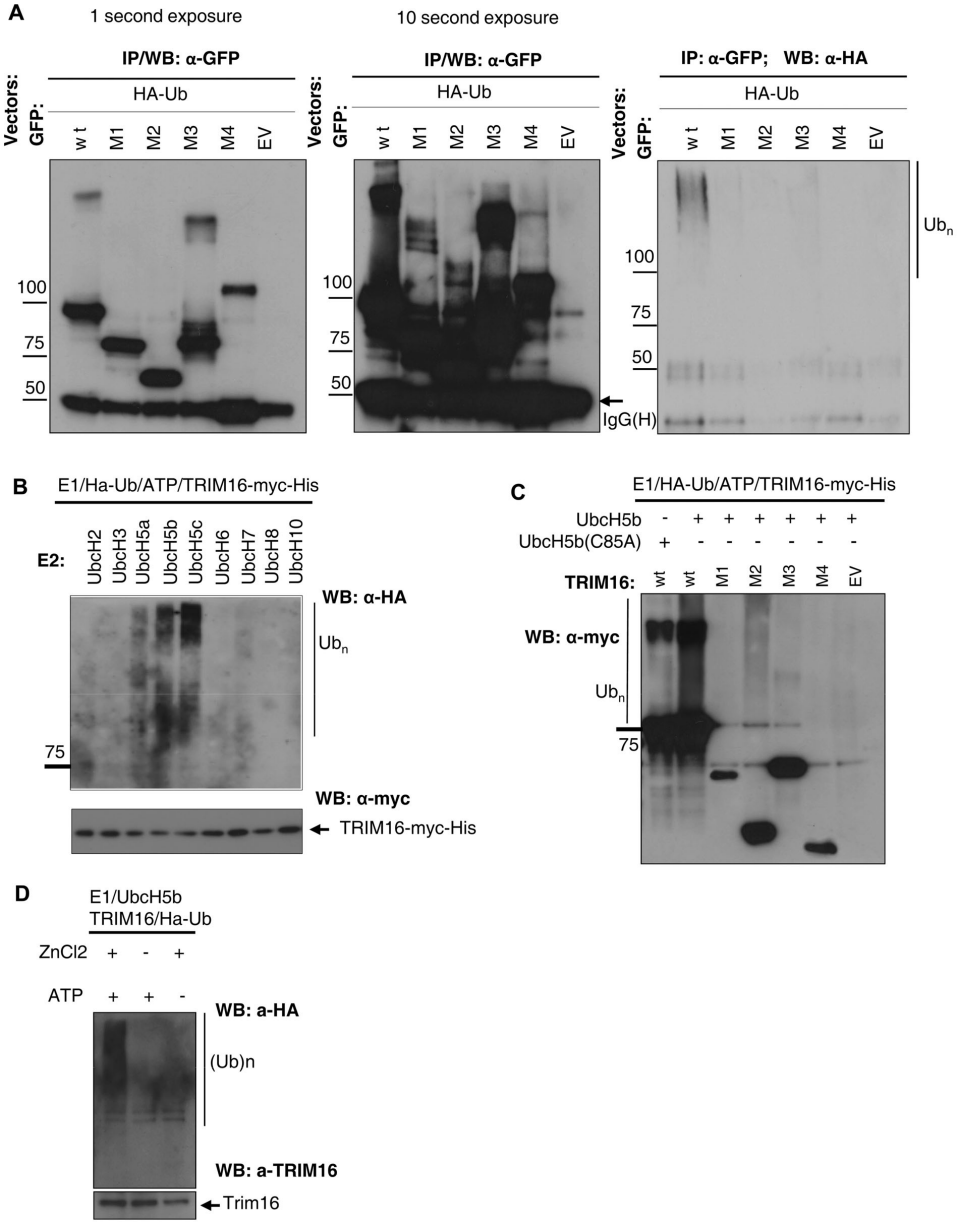
B-boxes are required for TRIM16’s E3 ligase activity. (A) TRIM16 *in vivo* polyubiquitination assay. In HEK293 cells, HA-Ub was co-transfected with various TRIM16-GFP domain deletion expression plasmids. The protein lysate was subjected to immunoprecipitation by GFP antibody, and subjected to Western blot and probed with anti-HA antibody for Ub (right panel) and anti-GFP antibody for TRIM16 (left and middle panel). Two exposure times are shown. GFP antibodies detect both un-ubiquitinated and polyubiquitinated forms of TRIM16. Polyubiquitinated smear is present in the sample transfected with wild-type TRIM16 and shown by anti-GFP and anti-HA antibodies. (B) *In vitro* ubiquitination assay with myc-His tagged TRIM16 together with a panel of E2 enzymes, showing activity with the UbcH5 family. (C) *In vitro* ubiquitination assay with full-length TRIM16, TRIM16 domain deletion mutants or empty vector showing extensive polyubiquitination with full-length TRIM16 as detected by Western blot with anti-myc antibodies. Numbers indicate protein size in kDa. (D) Recombinant TRIM16 (Abnova) was evaluated for E3 activity in the presence of recombinant E1, UbcH5b, and HA-Ub as indicated. The capacity to catalyse auto-ubiquitination *in vitro* was observed only in the presence of ZnCl_2_ and ATP. Western Blot (lower panel) with TRIM16 antibody showed amount of TRIM16 protein in each lane.

Protein ubiquitination occurs in a series of reactions in which a molecule of ubiquitin is transferred from the activating enzyme (E1), to a conjugating enzyme (E2), and, with the involvement of an E3 ligase, to a substrate or to the E3 ligase itself. Common features of E3 ligases are their ability to auto-ubiquitinate and self-degrade. TRIM16 is itself an unstable protein, degraded by proteasome mediated degradation. In various cell lines, TRIM16 protein quantity was increased after the proteasome inhibitor MG132 treatment (Figure 4A). Furthermore, TRIM16 has a short protein half-life around 4 hours measured by cycloheximide-based protein chase experiments in both SH-SY5Y and HEK293 cells (Figure 4B).

This result, together with the fact that TRIM16’s B boxes can adopt a RING-like structure moved us to investigate whether TRIM16 exhibits E3 ubiquitin ligase activity catalyzing auto-ubiquitination. TRIM16 E3 ligase activity was first examined by *in vivo* ubiquitination assay. TRIM16-GFP and TRIM16 domain deletion mutants (Figure 1A) and HA-tagged ubiquitin (Ub) were expressed in HEK293 cells; The cells were treated with MG132 four hours prior harvesting to preserve the polyubiquitin chains. GFP-tagged proteins were immunoprecipitated and the presence of polyubiquitinated TRIM16 was detected by Western blot. Only full-length TRIM16 has a high-molecular-weight smear, representing polyubiquitinated TRIM16 as detected by anti-GFP and anti-HA antibodies (Figure 5A).

TRIM16 E3 ubiquitin ligase activity was further examined by *in vitro* reaction. TRIM16-myc-His was incubated *in vitro* together with recombinant human E1, a panel of E2 enzymes, HA-tagged Ub and ATP. TRIM16 showed auto-polyubiquitination activity with a specific family of E2 enzymes, UbcH5 (UbcH5a, UbcH5b, UbcH5c) (Figure 5B). Other E2 enzymes tested produced no auto-polyubiquitination. To test whether B-Boxes of TRIM16 are required for auto-polyubiquitination, myc-His tagged TRIM16 and TRIM16 domain deletion mutants were incubated as above with E1, UbcH5b or catalytically inactive UbcH5b (C85A) enzymes. Extensive high molecular weight smears representing polyubiquitinated TRIM16, as detected by Western blot with anti-myc antibodies, is observed in the presence of catalytically active UbcH5b and wild-type TRIM16. In reaction where wild-type UbcH5b was substituted with a catalytically inactive variant UbcH5b (C85A), this auto-polyubiquitination was abolished (Figure 5C). To investigate whether recombinant TRIM16 synthesized using cell free system is able to auto-ubiquitinate *in vitro*, we have performed an *in vitro* ubiquitination assay. Recombinant TRIM16 purified from wheat germ extract was incubated *in vitro* together with recombinant human E1, UbcH5b, HA-tagged Ub and ATP; reaction was supplemented with zinc chloride (Zn) as described in Takahashi H *et al*, 2009 [22]. High-molecular-weight TRIM16 was detected when Zn and ATP were present in the assay (Figure 5D, lane 1).

The RING domain is typically known for its E3 ubiquitin ligase potential. Here we have demonstrated for the first time that a protein devoid of a classical RING domain can bind ubiquitin and auto-ubiquitinate via its B-boxes and function as an E3 ligase. In this report, we provide a first clue as to the function of the TRIM16 protein which is devoid of a classic RING domain. A surprising result of our study is that the B-boxes of TRIM16 exhibit an E3 ligase activity. We have also found that TRIM16 can homo and hetero-dimerize, a common feature found in other TRIMs. Therefore, in addition to its role as an E3 ligase we cannot exclude the possibility that TRIM16 may also control the stability of other TRIM proteins or that TRIM16 may function in a multi-subunit complex with other TRIMs enhancing their E3 ligase activity.

The proteasome degradation pathway remains one of the most powerful regulatory systems in cells of normal and malignant phenotypes. In recent years, an important function of TRIM proteins in mediating the transfer of ubiquitin to substrates as well as to themselves (auto-ubiquitination) have been shown to be involved in a number of critical processes in cell signalling, survival pathways and differentiation. Therefore, the interactions of the TRIM proteins have far reaching implications in all aspects of cells. Due to the scope of TRIM16 protein binding and structure studies as outlined here, our work suggest that TRIM16 is a useful biological model for the study of TRIM protein and ubiquitin interactions in the absence of the RING domain. Although TRIM16 lacks a RING domain, it nonetheless possesses important ubiquitination and heterodimerization functions, the implication of these functions in normal and cancer cells will need to be further elucidated.

## Materials and Methods

### Cell Culture and Conditions

BE2C, SHEP and SH-SY5Y cell lines were gifted by Dr. J. Biedler (Memorial Sloan-Kettering Cancer Center, New York). The human embryonic kidney 293 cells (HEK 293) were purchased from the American Type Culture Collection. All cells were cultured at 37°C in 5% CO_2_ in Dulbecco’s modified Eagle’s medium supplemented with L-glutamine and 10% fetal calf serum. For TRIM16 stability experiments, 30 µM of MG132 (Biomol, USA) was used in complete culture medium for 4 hours and 100 µg/ml cycloheximide (Biomol) for incubation of specified times.

### Transient Transfection

Transfections were carried out for 8 hours using Lipofectamine 2000 (Invitrogen, CA) according to the manufacturer’s instructions. Cells were harvested at 36 hours post-transfection or 28 hours for ubiquitination assays.

### Expression Vectors

The TRIM16 full-length cDNA was subcloned into the pcDNA3.1(-)/myc.His vector at the EcoRI multi-cloning site. The TRIM16-GFP deletion mutants were produced by OriGene (Rockville, MD, USA). The deletion mutants were synthesized by PCR of full-length TRIM16 cDNA (NM_006470.3). The final mutants were in the pCMV6-AC-GFP vector. In Figure 1A, the mutant products correspond to the following amino acid (AA) sequences; AA 1–564 (full-length), AA 161–564 (M1), AA 290–564 (M2), AA 1–372 (M3), AA 1-165 (M4) and AA 1-294 (M5) [3], [5].

### Western Blotting and Immunoprecipitation (IP)

Anti-TRIM16 (Bethyl Laboratories, TX), anti-TurboGFP (Evrogen, Moscow), anti-Actin (Sigma, Sydney), and anti-myc tag Abs (Cell Signalling Technology, MA) were used in Western blots. Anti-His tag (Cell signaling, USA), anti-HA tag (Cell signaling, USA) and anti-PML (Santa Cruz Biotechnology, USA) antibodies were used in IP assays. For IP studies, cells were lysed in NP-40 buffer with 150 mM NaCl and protease inhibitors (Roche, Sydney). Lysates containing 0.5 mg of protein were immunoprecipiated with 1 µL of specified Abs.

### In Vivo Ubiquitination Assay

Cells were transfected with GFP/myc tagged TRIM16/mutant and HA-Ub expression plasmid for 24 hours following 4 hours incubation with the proteasome inhibitor MG132 (Biomol, USA) to preserve the polyubiquitin chain and then lysed under denaturing conditions. 2 mg of protein was immunoprecipiated with specified antibodies. After immunoprecipitation, the samples were washed and resolved by SDS-PAGE and analysed by western blot using anti-HA antibody.

### In Vitro Ubiquitination Assay

TRIM16-myc-His, expressed in HEK293 cells was precipitated using myc-tag Ab and GammaBind G sepharose beads (GE Healthcare, USA), beads were extensively washed and incubated for 90 minutes at 32°C in *In Vitro* Ubiquitination Buffer (25 mM Tris-HCl pH 7.6, 5 mM MgCl_2_, 100 mM NaCl) containing 100 ng of E1, 150 ng of E2 enzymes (Boston Biochem, MA), 5 µg of HA-Ub (Boston Biochem, MA), 2 mM ATP (Sigma Aldrich) and 2 mM DTT (Sigma Aldrich). After incubation, beads were extensively washed and subjected to Western blotting. *In vitro* ubiquitination assay containing 500 ng of recombinant TRIM16 (Abnova) and 10 µM ZnCl_2_ was performed as described above.

### Computational Modeling

The amino acid sequence of the whole human TRIM16 was threaded onto the top 10 protein structure templates predicted using HHpred on the Max Plank Bioinformatics Server (http://toolkit.tuebingen.mpg.de/) and a homology model was constructed using MODELLER (http://salilab.org/modeller/) [10]. The solution structures of MID1 B1B2 boxes were downloaded from the Protein Data Bank (PDB) [11]. The top 10, of the 20 solution structures of MID1 were aligned via its α-carbons with the B-box model of TRIM16 in Pymol [12]. Zinc ions in TRIM16 were positioned in the same location as found in MID1 using Sybyl-X1.1. This model was then minimized under the MMFFs force field in Sybyl-X1.1 until energy convergence was reached (58043 iterations).
